# Avoidance of seismic survey activities by penguins

**DOI:** 10.1038/s41598-017-16569-x

**Published:** 2017-11-24

**Authors:** Lorien Pichegru, Reason Nyengera, Alistair M. McInnes, Pierre Pistorius

**Affiliations:** 1 DST/NRF Centre of Excellence at the Percy FitzPatrick Institute for African Ornithology, Institute for Coastal and Marine Research and Department of Zoology, Nelson Mandela University, Port Elizabeth, South Africa; 2 Present Address: Seabird Division, BirdLife South Africa, Cape Town, South Africa

## Abstract

Seismic surveys in search for oil or gas under the seabed, produce the most intense man-made ocean noise with known impacts on invertebrates, fish and marine mammals. No evidence to date exists, however, about potential impacts on seabirds. Penguins may be expected to be particularly affected by loud underwater sounds, due to their largely aquatic existence. This study investigated the behavioural response of breeding endangered African Penguins *Spheniscus demersus* to seismic surveys within 100 km of their colony in South Africa, using a multi-year GPS tracking dataset. Penguins showed a strong avoidance of their preferred foraging areas during seismic activities, foraging significantly further from the survey vessel when in operation, while increasing their overall foraging effort. The birds reverted to normal behaviour when the operation ceased, although longer-term repercussions on hearing capacities cannot be precluded. The rapid industrialization of the oceans has increased levels of underwater anthropogenic noises globally, a growing concern for a wide range of taxa, now also including seabirds. African penguin numbers have decreased by 70% in the last 10 years, a strong motivation for precautionary management decisions, including the exclusion of seismic exploratory activities within at least 100 km of their breeding colonies.

## Introduction

Marine seismic surveys explore subterranean geological features for petroleum, natural gas and mineral deposits, and produce the most intense man-made ocean noise^1^, that together with commercial shipping, sonar systems and blasting have altered the ocean environment^2^. Seismic survey operations utilize air guns towed at a depth of 4–8 m that emit sharp, loud sounds directed at the sea floor in the range 230–255 dB re 1 µPa at 1 m, generally at low frequencies of 10–100 Hz^3^, although there is an increased interest in using higher frequencies, above 1 kHz^4^. The acoustic energy is directed towards the seabed, but considerable energy is propagated horizontally, generally detectable up to 50–75 km from the sound source in shallow waters^2^ and up to 4000 km in deep waters^5^. Some seismic operations can extend over large areas (>50 000 km^2^ ^3^,) and can operate continuously for months^6^. With the ever increasing demand on energy in recent years, both the frequency and total area surveyed by seismic activities has dramatically expanded^7^, with impacts on marine fauna of growing concern^8^.

Many marine animals, from invertebrates to cetaceans, use underwater sounds for crucial biological activities such as foraging, orientation, communication, predator avoidance, mate selection, individual recognition or parent-offspring bonding^9^. Much of the research on the impacts of seismic surveys has focused on marine mammals, revealing changes in diving patterns^10^, increased calling activity^11^, hearing impairments^2^, habitat displacement^12^, and possibly lethal bends (i.e. sound-induced growth of gas bubbles in super-saturated tissues of diving mammals^13,14^). This series of issues prompted the Joint Nature Conservation Committee (JNCC) to establish guidelines to minimise the impacts of seismic operations on cetaceans^15^, currently adopted in various parts of the world^16^. These include the mandatory assignment of marine mammal observers on seismic survey vessel and the use of “soft-starts”, where power levels of airguns are slowly built up to operational levels over at least 20 minutes, “to give adequate time for marine mammals to leave the vicinity”^15^. Recent evidences however suggest these requirements may not be sufficient, as observers sometimes lack adequate training, or may have limited power over the action of the vessel in some companies, while soft-starts assume that animals can, and are willing to, move away from the disturbance, which is not necessarily the case^17,18^.

Other less conspicuous taxa are also affected by underwater noises and seismic operations (e.g.^19^). A recent review highlighted impacts on physical, behavioural and physiological aspects of some fish and invertebrates^20^. For example, seismic surveys may cause barotrauma in fish (i.e. damage of tissues and organs due to rapid changes in pressure^21^) and increase mortality of fish eggs^22^. Loud underwater sounds can damage sensory cells in fish ears (e.g.^23^) and the statocysts of squids, possibly leading to lethal acoustic trauma^24,25^. Several fish species have been shown to descend to greater depths in response to seismic activities^26^, with reduced foraging efficiency in some instances^27^. However, the results of these studies are contradictory at times, depending on the intensity of the sound tested, its proximity to the study species, and whether the study species were free-ranging or in a controlled environment^20^. Nonetheless, elevated mortality in zooplankton has been demonstrated following exposure to seismic gun arrays in an area of up to 1.2 km radius of the activity, with potential negative impacts on ocean ecosystem function^28^.

By contrast, there is no evidence to date on the potential effects of these surveys on seabirds. In particular, flightless birds such as penguins, due to their largely aquatic existence, are expected to be sensitive to loud sounds underwater^29^. Penguins are among the most threatened bird families, largely due to the negative effects of habitat change associated with human activities, such as oil pollution, competition with fisheries and climate change^30^.

African penguins (*Spheniscus demersus*) are endemic to southern Africa, with their population having recently decreased by 70% since 2004^31^. This has raised grave concern about impacts of anthropogenic disturbances on land and at sea on the future viability of this species. To our knowledge, there is no information about the impacts of underwater sound to African penguins, although previous observations reported strong impact of blasting on southern rockhoppers (*Eudyptes chrysocome*) and African penguins, which were found floating unconscious close to blast sites at sub-Antarctic Marion Island and Saldhana Bay, South Africa^32,33^, respectively. African penguins can hear sounds between 100 and 15 000 Hz^34^, well within the range of seismic survey operations. They dive 30 m deep on average, with dives lasting up to 4 minutes^35^, within 30–40 km of their colonies when breeding^36^. They are therefore limited in their capacity to avoid influences of seismic activity in close proximity to their breeding sites. Having been recently upgraded to endangered^37^, major conservation efforts are currently being implemented with the intention of bolstering African penguin numbers, and known threats to this species are currently closely monitored^38^. In this study, we assessed the foraging behaviour of African Penguins before, during and after seismic operations conducted within 100 km of their two largest breeding colonies, both situated in Algoa Bay, home to approximately half of their global population^31^.

## Results

In total, 333 complete individual foraging tracks were recorded from Bird and St Croix islands between March and May 2009–2013 from breeding African penguins during and outside seismic activities at St Croix Island (n = 31 and n = 74 respectively) and Bird Island (n = 20 and n = 208 respectively).

**Figure 1:**
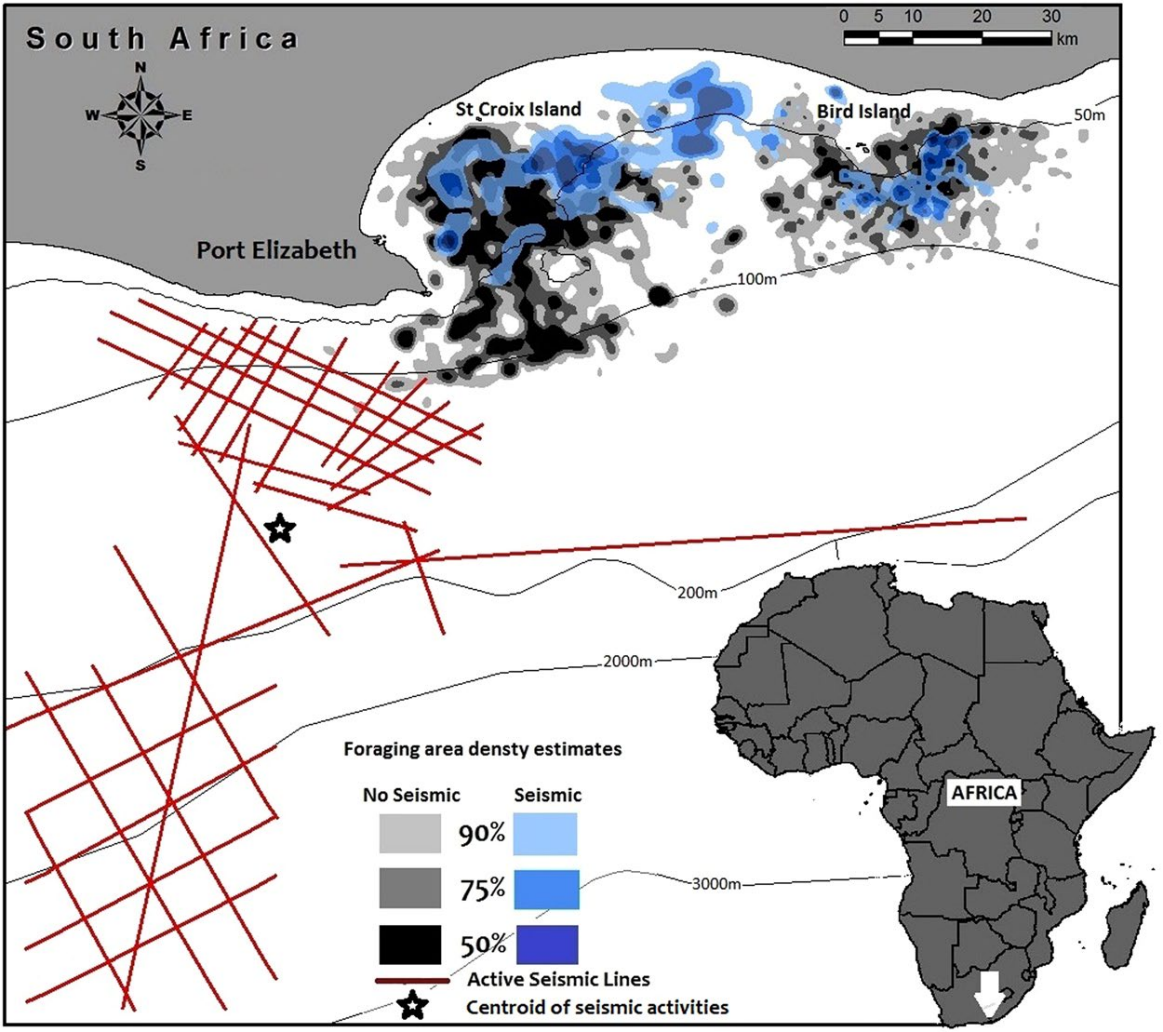
Overlay of African penguin foraging area estimates based on 50%, 75%, and 90% utilisation distribution contours created using kernel density estimates of foraging tracks outside (2009–2013, grey shades) and during (March 2013, blue shades) seismic activities. Concurrent seismic operations in March 2013 are shown with red lines and the centroid of the activities is also shown. The map was produced using ArcGIS 10.4 (http://desktop.arcgis.com/en/arcmap/10.4).

**Figure 2:**
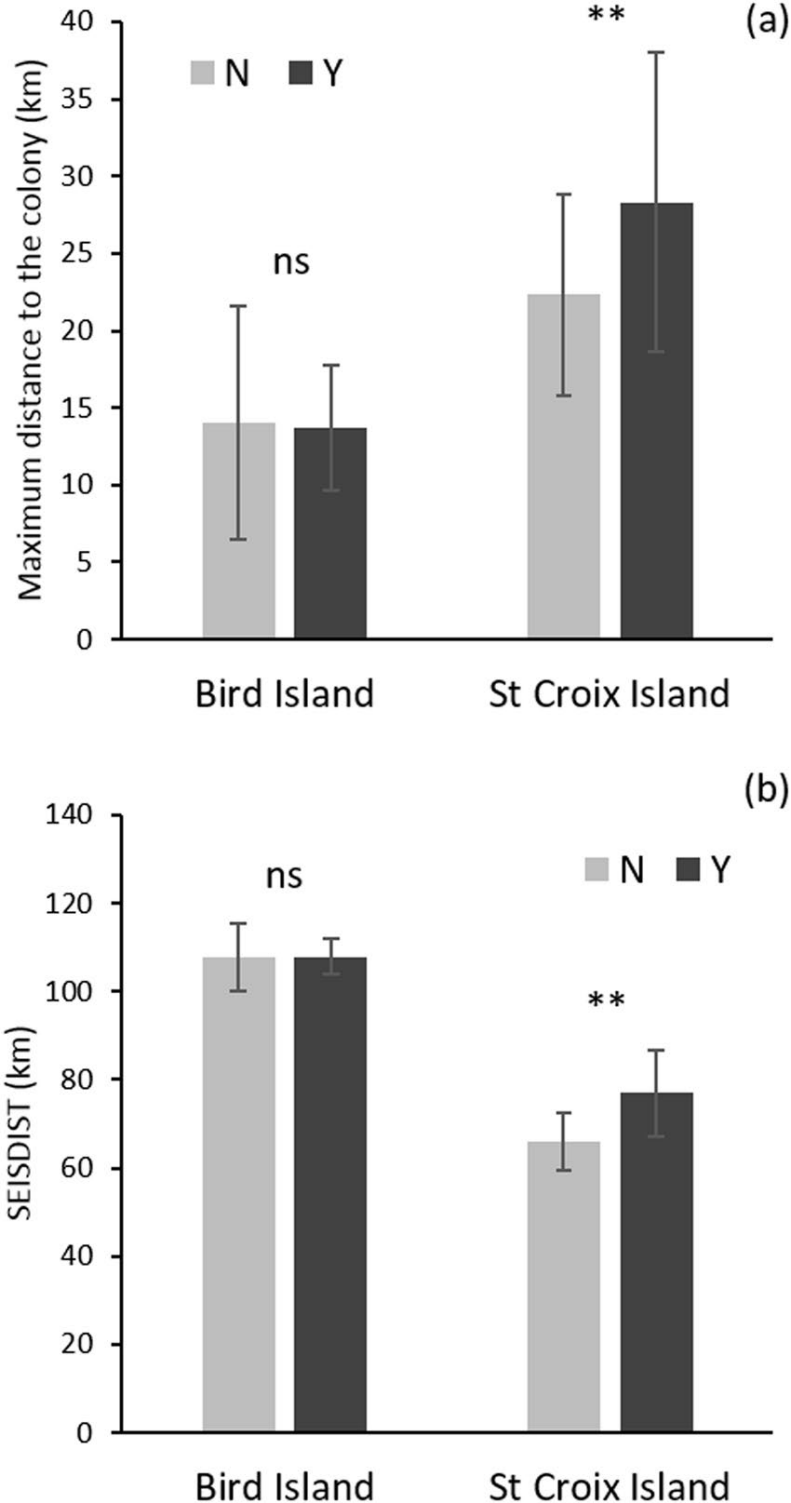
Mean ± SD maximum foraging distance from the colony (**a**) and distance between the location of a penguin fix at the maximum distance from its colony to the centroid of seismic activity (SEISDIST) (**b**) of birds breeding on Bird and St Croix islands in March-May 2009–2013, outside (N) and during (Y) seismic activity.

Foraging effort (duration of trip at sea, foraging path length, maximum distance from the colony) varied among years and was generally greater for birds from St Croix Island ((Table 1, Fig. 1). Compared to other years, foraging effort was slightly lower in 2013 in the absence of seismic activity but increased for penguins from both colonies when the seismic survey was taking place (Table 1). Maximum foraging distance from the colony increased significantly for St Croix birds during seismic activities (p = 0.007, Table 2, Fig. 2a).

**Table 1:** Mean ( ± SD) foraging effort (trip duration, path length and maximum distance to the colony) and distance between the location of penguin fixes at the maximum distance from the colony to the centroid of seismic activity (SEISDIST) for birds breeding on St Croix and Bird islands between March – May 2009–2013 outside (N) and during (Y) periods of seismic activity.

	Trip duration (h)		Path length (km)		Max. distance (km)		SEISDIST (km)	
	St Croix	Bird	St Croix	Bird	St Croix	Bird	St Croix	Bird
2009 (N)	16.4 ± 4.0	18.0 ± 6.3	47.9 ± 17.6	41.0 ± 8.0	19.7 ± 7.2	12.8 ± 4.5	65.2 ± 7.2	112.7 ± 6.9
2010 (N)	25.7 ± 6.0	19.6 ± 8.3	68.4 ± 11.8	49.3 ± 25.7	25.5 ± 5.8	13.1 ± 4.3	65.5 ± 16.0	107.8 ± 9.8
2011 (N)	20.5 ± 4.4	20.9 ± 10.1	66.1 ± 14.1	41.4 ± 18.2	22.4 ± 7.1	16.1 ± 11.2	64.1 ± 10.7	104.6 ± 16.5
2012 (N)		19.4 ± 4.5		55.9 ± 10.6		15.8 ± 8.0		109.4 ± 13.5
2013 (N)	17.8 ± 2.9	13.6 ± 5.3	61.5 ± 10.3	39.8 ± 15.6	22.5 ± 5.6	12.1 ± 4.6	67.9 ± 11.7	106.6 ± 7.3
2013 (Y)	19.3 ± 3.7	14.2 ± 3.3	67.6 ± 13.6	43.3 ± 11.9	28.3 ± 9.7	13.7 ± 4.0	77.0 ± 13.6	107.9 ± 7.4

**Table 2:** Coefficients (β) and standard errors (SE) of general linear models fitted to assess the influence of seismic activity on four responses, three path metrics (trip duration, path length and maximum distance to the colony) and the distance between the location of penguin fixes at the maximum distance from the colony to the centroid of seismic activity (SEISDIST) for birds breeding on St Croix and Bird islands.

Explanatory variables	Trip duration		Path length		Max. distance		SEISDIST	
	β (SE)	p	β (SE)	p	β (SE)	p	β (SE)	p
**St Croix Island**								
Seismic activity (Y)	0.08 (0.07)	0.26	0.09 (0.08)	0.21	**0.23 (0.08)**	**0.007**	**0.13 (0.05)**	**0.008**
Year (2010)	**0.45 (0.09)**	**<0.001**	**0.36 (0.1)**	**<0.001**	**0.26 (0.12)**	**0.04**	0.01 (0.07)	0.95
Year (2011)	0.23 (0.07)	0.002	**0.32 (0.08)**	**<0.001**	0.13 (0.11)	0.23	−0.02 (0.06)	0.77
Year (2013)	0.09 (0.07)	0.25	**0.25 (0.08)**	**0.002**	0.14 (0.1)	0.18	0.04 (0.06)	0.47
**Bird Island**								
Seismic activity (Y)	0.05 (0.11)	0.68	0.09 (0.09)	0.36	0.13 (0.13)	0.32	0.01 (0.03)	0.66
Year (2010)	0.09 (0.1)	0.41	0.09 (0.11)	0.42	0.03 (0.13)	0.86	−0.04 (0.03)	0.11
Year (2011)	0.15 (0.09)	0.12	0.01 (0.11)	0.92	0.23 (0.12)	0.06	**−0.07 (0.02)**	**0.003**
Year (2012)	0.08 (0.11)	0.48	**0.31 (0.11)**	**0.004**	0.21 (0.13)	0.1	−0.03 (0.03)	0.23
Year (2013)	**−0.28 (0.09)**	**0.002**	−0.03 (0.1)	0.76	−0.06 (0.12)	0.65	**−0.05 (0.02)**	**0.03**

Over the entire study period, St Croix Island penguins generally foraged towards the south east of their colony, or due south mostly within the 100 m bathymetric contour of the continental shelf (Fig. 1). Therefore, their preferred foraging areas were closer to where the seismic survey vessel was located in 2013 compared to that of Bird Island birds (ca 65 km on average versus >100 km for St Croix and Bird islands respectively, Table 1, Fig. 1). When seismic activities took place in March 2013, St Croix birds switched to foraging due east or north east of their colony (Fig. 1), constituting a significant change in bearing (Watson 2-sample test = 0.47, p < 0.001). As a result, the birds foraged significantly further away from the centroid of the seismic activities during that period (77 km, compared to ca 65 km on average in the absence of seismic activity, p = 0.008, Fig. 2b, Table 2). By contrast, Bird Island penguins consistently travelled due east to south-southwest of their colonies, also within the 100 m bathymetric contour of the continental shelf (Fig. 1), regardless of seismic activities. Thus, there was no significant change in bearing for birds from Bird Island (Watson 2-sample test = 0.14, p > 0.1).

Comparing penguin’s foraging effort within 2013 only, once the seismic operations ceased the maximum distance travelled by St Croix penguins significantly decreased (Table 1, Mann Whitney U test, w = 258.5, p = 0.03), as well as their foraging distance to the centroid of the positions of seismic vessel (SEISDIST, Table 1, w = 254, p = 0.02). Trip duration and foraging path length remained similar (w = 97, p = 0.2; w = 138, p = 0.17, respectively).

## Discussion

Penguins foraging <100 km from active seismic operations showed a clear change of foraging direction during seismic periods, increasing their distance between their feeding area and the location of the seismic vessel. To our knowledge, this is the first record of avoidance behaviour by a seabird to sounds generated from anthropogenic activities at sea. African penguins from St Croix Island seemed to have avoided airgun sounds by foraging east of their colony, diverting from their traditional feeding grounds located in a more southerly direction. Avoidance behaviour as a response to seismic operations has been documented in many cetaceans (see^13^ for a review). For example, bowhead whales *Balaena mysticetus* avoided the area of seismic sources by >20–30 km and showed signs of altered diving and surface behaviour at distances up to 73 km from seismic vessels^39^. Similarly, avoidance behaviour by gray whales *Eschrichtius robustus* were recorded at ranges up to 24 km from seismic activity and altered behaviour (faster and straighter swimming and shorter blow intervals during seismic noise) at ranges >30 km^40^. Humpback whales *Megaptera novaeangliae* showed avoidance behaviour at a range of 5–8 km from a full-scale seismic array and maintained a stand-off range of 3–4 km^12,18^. Avoidance behaviour has also been noted in fish, although behavioural studies on unrestrained fish exposed to airgun sounds are scarce (see^20^ for a review).

The avoidance behaviour by penguins observed in this study may be explained by either a direct disturbance from the noise generated by the operation or a change in fish distribution during that period (possibly as a result of seismic activities). The present study cannot disentangle the two effects. A possible decrease in prey availability following seismic operations was previously raised as a cause of concern as an indirect impact of surveys on marine mammals^13^. Incidences of reduced commercial fish catches have been recorded in areas where seismic survey were active or directly after the cessation of activities, suggesting avoidance of the area by the targeted fish species (e.g.^22^). However, several *in situ* studies showed limited direct response of fish to seismic activities^20^, and when there was a response, the vertical rather than the horizontal distribution of fish was generally influenced (e.g.^26,41^). Consequently, reduced commercial catch rates associated with seismic activity may possibly have resulted from a vertical displacement of fish. Small-scale acoustic fish surveys assessing distribution and abundance of small pelagic fish in Algoa Bay around both penguin colonies^42^ did not show a significant change in distribution and/or abundance of small pelagic fish in the region in March 2013 compared to a few months prior to or after the seismic operations^43^. Therefore, African penguins likely relocated away from their traditional feeding zone to avoid the disturbance generated by the noise of the seismic vessels, rather than to follow their prey.

The exposure to intense sounds, such as the shooting of airguns during seismic operations, can adversely affect the hearing capacity of marine mammals and other species, either temporarily or permanently^1^. This impairment can reduce individual foraging performance, by diminishing prey detection capabilities, but also indirectly by reducing their ability to detect predators or assess their environment, thereby reducing the overall fitness of the individuals affected^44^. Such threshold shifts have been demonstrated experimentally in several species of fish and invertebrates, either in the laboratory or in cages placed in the wild (see review in^20^) but are generally difficult to assess in wild populations. The hearing capabilities of birds are complex and poorly understood^45^. Although some information is available on underwater hearing capacities of cormorants^46^, virtually no research has been conducted on hearing in penguins in particular^47^. The impact of noise on terrestrial birds is, however, well known and noisy anthropogenic activities can reduce the abundance of passerines, although the mechanisms are unclear^48^. A potential cause could be related to interference with vital life histories involving acoustic communication, such as mate selection or territorial defense, which may ultimately affect breeding success^48^. At sea, however, such mechanisms are unlikely. Loud underwater sounds, such as airgun shooting, may be uncomfortable for birds, especially as sounds travel five times faster in water than in air and cover much greater distances at higher amplitude levels. Pingers emitting sounds of 1 kHz at 120 dB attached to driftnets significantly reduced by-catch of common murres *Uria aalge* ^49^, although the study could not establish if the sounds emitted by the pingers were repulsing birds or their prey. African penguins are known to be sensitive to sounds as low as 100 Hz^34^, therefore it is possible that the sounds emitted by the surveys were a direct disturbance to them.

Noises from seismic operations may also have disrupted communication between African penguin individuals and groups, leading to a change in foraging behaviour, especially considering that the fundamental frequency (i.e. the lowest frequency component) of their vocalisations is around 250 Hz and plays a key role for individual discrimination^50^. A number of marine top predators rely on acoustic signaling for communication, orientation, locating prey and predators^2^. While knowledge of their use of vocalisation for communication at sea remains very limited, it is known that penguins use sound extensively on land for intraspecific communication including mate and chick recognition^51,52^. Contact calls have been primarily recorded for penguins at the surface when at sea [refs^51,53^, McInnes unpubl.]. Lessening an individual’s ability to detect socially relevant signals could affect biologically important processes (e.g.^6,13^). African penguins often forage in groups^54^, which improves their prey capture efficiency^55^. It is therefore possible that they may use acoustic signals to coordinate their movement at sea and may be disturbed by loud anthropogenic activities. African penguins are also known to respond to underwater vocalisations of predators^56^. Anthropogenic noise pollution may therefore also affect their capacity to detect the presence of a predator, with potential negative consequence on their survival.

African penguins quickly reverted to normal foraging behaviour after cessation of seismic activities during this study, which suggest a relatively short-term influence of seismic activity on these birds’ behaviour and/or that of their prey. Most bird and many fish species have the capacity to regenerate lost or damaged sensory cells of the ear^57^, although we cannot rule out potential longer-terms impacts on their hearing ability. Longer or repeated exposure to elevated underwater noise levels can affect reproductive and growth processes in some marine organisms^58^ and lead to chronic stress^59^, which in turn can lead to a depressed immune function^60^. The potential for disturbance from cumulative impacts is particularly high for resident species with limited dispersal abilities^7^. This might be particularly true for African penguins breeding on St Croix Island, the largest African penguin colony^61^, as it is located in the vicinity of two large industrial harbours in the bay.

The biological significance of altered behaviours during seismic surveys remains difficult to measure. Some behavioural responses have been associated with reduced rate of foraging or of predator avoidance (e.g.^27^), others with increased energy expenditure (e.g.^62^). African penguins increased their foraging effort during seismic periods, particularly when their general foraging area was <100 km from the seismic operations. Increasing energy expenditure at sea to locate food can negatively affect penguins’ reproductive output^63^. Breeding success of African penguins is currently at very low levels due to a suite of threats, from predation to extreme weather events^64^, and reduced food availability due to local competition with fisheries^37,65^. As long-lived species, biologically important changes in rates of population trends are difficult to identify, particularly over a short time scale. Following of a recent drastic decrease in their population numbers^61^, a Biodiversity Management Plan has been drafted by the South African Department of Environmental Affairs^38^, to assess and manage the threats to African penguins. Relevant to this management plan, results of the current study demonstrate that seismic survey operations may negatively impact penguins within 100 km of their feeding localities, and should be restricted to areas >100 km from African penguin colonies.

Rapid industrialization associated with resource extraction in the oceans has increased levels of underwater anthropogenic noises, a growing concern for the survival of a wide range of taxa^1,21^. In addition to over-fishing, habitat destruction and chemical pollution, underwater noise pollution is now recognized as a significant threat to marine wildlife^19^. Many underwater animals from invertebrates to marine mammals, rely on sound-based cues to forage, attract a mate or avoid predation^1,66–68^. Therefore anthropogenic sounds may perturb crucial life history traits^21^. Direct evidences for impacts of noise pollution on marine wildlife remain scarce (e.g.^28^), even if deemed very likely^14,69^. This is largely due to the difficulty in acquiring the necessary data to demonstrate such effects, despite their potential negative impact on another major marine economic sector, commercial fishing (e.g.^70,71^). Consequently, the existing evidences are largely anecdotal (e.g.^24,72^) and there is a crucial need for additional studies of impact of loud noises, such as generated by oil and gas exploration activities, on hearing capabilities, avoidance behaviour and prey dynamics of animals including seabirds. Penguins are currently the most threatened seabird family, and based on the findings of this study, prudent planning of seismic exploration surveys in their habitat is required^7^.

## Methods

### Foraging behaviour data collection

The foraging behaviour of adult African penguins raising chicks of 1–3 weeks old was studied in Algoa Bay at Bird Island (33° 50’ S,26° 17’ E) and St Croix Island (33° 48’ S, 25° 46’ E), between 2009 and 2013. This dataset is part of a long-term monitoring project (e.g.^34^) and only data from March to May were selected for this study, to control for possible behavioural differences outside this period driven by changing environmental conditions during the austral winter (Pichegru & McInnes unpubl. data). All methods were approved by South African National Parks (PICL578), the South African Department of Environmental Affairs (res2013–05) and with ethic clearances from University of Cape Town (2009/V2/LP) and Nelson Mandela Metropolitan University (NMMU-A15-SCI-ZOO-008). Methods were performed in accordance with the relevant permits and regulations. Sampling the behaviour of the penguins took place in four consecutive years (2009–2012) when there was no seismic activity; in March 2013 concomitantly with seismic surveys, and in April-May 2013 after the operations ceased. African penguins were equipped with GPS loggers (earth & OCEAN Technologies™, Germany, or CatTrack™, USA) recording location every minute at an accuracy of <10 m, and weighing <2.5% of adult body mass. Birds were caught at their nest site, the loggers were attached to their lower back feathers with waterproof tape, and they were released at the nest within <6 min (see details in^35^). Nest sites were then monitored until the birds returned and the devices were removed. If several foraging tracks were recorded per individual bird, only one (the first one recorded) was included in the analyses to avoid pseudo-replication.

### Seismic sound source

Seismic surveys (2D) took place in South Africa in the Algoa Bay/Gamtoos river mouth area from 15th of February to 22nd of March 2013, covering an estimated distance of 1 527 km and a total area of 6 700 km^2^ (Fig. 1). Airguns were shot at point intervals of 25 m at an average of 169 airgun shots per hour. Acquisitions were done 24 hours a day, at an average of 11 acquisitions per hour. The source was made up of 4 sub-arrays of airguns (Bolt Long Life 1 900 XT) with a total volume of 4 230 in³ at 2 000 psi ± 10% pressure for the array. Data acquisition was only paused in the event of the presence of marine mammals in close proximity of the ship and when changing lines.

### Statistical analyses

From the GPS tracks, we estimated foraging effort (i.e. foraging trip duration, foraging path length and maximum distance from the colony) and the distance of the furthest GPS position, i.e. location of maximum distance for each individual, to the centre of seismic activity (hereafter referred to as SEISDIST), which was determined by calculating the centroid of all georeferenced seismic activities in March 2013 (Fig. 1). Tracks were filtered to exclude erroneous fixes that exceeded the potential distance covered given their mean maximum speed (12.4 km h^−1^, ref.^73^). Trip duration was only calculated for tracks with start and end fixes <5 km from colonies and foraging path length for tracks that had gaps <2 h. When start and/or end fixes were not at the colony but within 5 km, distance travelled and duration were calculated from the average travelling speed of African Penguins in Algoa Bay (2.5 km h^−1^, ref.^74^). The bearing of individual tracks from the island to the farthest point of their trip was calculated in software R^75^ (R Core Team, 2015) using package ‘Geosphere’^76^. Kernel density analysis was performed in ArcGIS 10.4 on the totality of the individual tracks, using the adaptive kernel method with smoothing parameters selected based on least-squares-cross-validation. Estimates were created for foraging ranges based on 50, 75, and 90% utilisation distribution.

We used generalised mixed effects models (GLMM) with a Gamma error distribution and a log link function (‘lme4’ package^77^,) to assess the influence of seismic activity on penguin foraging effort and SEISDIST, with presence/absence of seismic activity and year as fixed effects. Year was included to account for annual variability in oceanographic conditions and fishing intensity, which are known to influence prey availability and penguin foraging performance in this region^33,34,41^. For the models using variables of foraging effort as responses we included colony as a fixed effect; for SEISDIST as the dependant variable models were fitted separately for each colony due to the bi-modal nature of the response. In addition, in order to establish if conditions potentially reverted back to ‘normal’ once the operations ceased, a within year effect was assessed for the significant foraging parameters, (maximum foraging distance and SEISDIST, see below) against seismic activity in 2013 only for St Croix penguins.

Non-parametric circular statistics using Watson’s two-sample test of homogeneity were used to assess the differences in bearing from penguins colonies to their maximum distance location with and without seismic activity. This was done for each colony separately using package ‘circular’ in R^78^.

### Data availability

The datasets generated during and/or analysed during the current study are available from the corresponding author on reasonable request.

## References

[1] Southall BL (2007). Marine mammal noise exposure criteria: initial scientific recommendations. Aquatic Mammals.

[2] Hildebrand JA (2009). Anthropogenic and natural sources of ambient noise in the ocean. Mar. Ecol. Progr. Ser..

[3] Richardson, W. J., Green, J. C. R., Malme, C. I. & Thomson, D. H. *Marine mammals and noise* (Academic Press, San Diego, 1995).

[4] Landro M, Amundsen L, Barker D (2011). High-frequency signals from air-gun arrays. Geophysics.

[5] Nieukirk SL (2012). Sounds from airguns and fin whales recorded in the mid-Atlantic Ocean, 1999–2009. J. Acoust. Soc. Am..

[6] Clark, C. W. & Gagnon, G. C. Considering the temporal and spatial scales of noise exposures from seismic surveys on baleen whales. International Whaling Commission Scientific Committee document SC/58/E9 (2006).

[7] Nowacek DP (2015). Marine seismic surveys and ocean noise: time for coordinated and prudent planning. Frontiers Ecol. Evol..

[8] Williams R (2015). Impacts of anthropogenic noise on marine life: publication patterns, new discoveries, and future directions in research and management. Ocean Coast. Manag..

[9] Au, W. W. L. & Hastings, M. C. *Principles of Marine Bioacoustics* (Springer, US, 2008).

[10] Robertson FC (2013). Seismic operations have variable effects on dive-cycle behavior of bowhead whales in the Beaufort Sea. Mar. Ecol. Progr. Ser..

[11] Clark CW (2009). Acoustic masking in marine ecosystems: intuitions, analysis, and implication. Mar. Ecol. Progr. Ser..

[12] McCauley RD, Jenner M-N, Jennei C, McCabe KA, Murdoch J (1998). The response of Humpback Whales (*Megaptera novaeangliae*) to offshore seismic survey noise: preliminary results of observations about a working seismic vessel and experimental exposures. Austral. Petrol. Prod. Explor. Assoc.

[13] Gordon J (2004). A review of the effects of seismic surveys on marine mammals. Mar. Technol. Soc. J..

[14] Cox TM (2006). Understanding the impacts of anthropogenic sound on beaked whales. J. Cetac. Res. Manage..

[15] JNCC (U.K. Joint Nature Conservation Committee). *Guidelines for minimising acoustic disturbance to marine mammals from seismic surveys* (Joint Nature Conservation Committee, Peterborough, UK, 1998).

[16] Compton R, Goodwin L, Handy R, Abbott V (2008). A critical examination of worldwide guidelines for minimising the disturbance to marine mammals during seismic surveys. Mar. Pol..

[17] Wright AJ, Cosentino M (2015). JNCC guidelines for minimising the risk of injury and disturbance to marine mammals from seismic surveys: we can do better. Mar. Poll. Bull..

[18] Dunlop RA (2016). Response of humpback whales (*Megaptera novaeangliae*) to ramp-up of a small experimental air gun array. Mar. Poll. Bull..

[19] Popper AN (2016). Effects of exposure to the sound from seismic airguns on pallid sturgeon and paddlefish. PLoS One.

[20] Carroll AG, Przeslawski R, Duncan A, Gunning M, Bruce B (2017). A critical review of the potential impacts of marine seismic surveys on fish & invertebrates. Mar. Poll. Bull..

[21] Popper, A. N. & Hawkins, A. D. *The Effects of Noise on Aquatic Life* (Springer, 2012).

[22] Turnpenny, A. W. H. & Nedwell, J. R. The effects on marine fish, diving mammals and birds of underwater sound generated by seismic surveys. Fawley Aquatic Research Laboratories Ltd., FCR 089/94:1–40 (1994).

[23] McCauley RD, Fewtrell J, Popper AN (2003). High intensity anthropogenic sound damages fish ears. J. Acoust. Soc. Am..

[24] Guerra Á, González ÁF, Rocha F (2004). A review of the records of giant squid in the north-eastern Atlantic and severe injuries in *Architeuthis dux* stranded after acoustic explorations. ICES CM.

[25] André M (2011). Low-frequency sounds induce acoustic trauma in cephalopods. Front. Ecol. Env..

[26] Slotte A, Hansen K, Dalen J, Ona E (2004). Acoustic mapping of pelagic fish distribution and abundance in relation to a seismic shooting area off the Norwegian west coast. Fish. Res..

[27] Voellmy IK (2014). Acoustic noise reduces foraging success in two sympatric fish species via different mechanisms. Anim. Behav..

[28] McCauley, R. D. *et al*. Widely used marine seismic survey air gun operations negatively impact zooplankton. *Nature Ecol*. *Evol*. 10.1038/s41559-017-0195 (2017). 10.1038/s41559-017-0195 28812592

[29] O’Brien, P. E. Impacts of marine acoustic technology on the Antarctic environment, SCAR Ad Hoc Group on marine acoustic technology and the environment, Version 1.2 (2002).

[30] Trathan PN (2015). Pollution, habitat loss, fishing and climate change as critical threats to penguins. Conserv. Biol..

[31] Crawford RJM (2015). A changing distribution of seabirds in South Africa – the possible impact of climate and its consequences. Front. Ecol. Evol..

[32] Brown CR, Adam NJ (1983). The effect of underwater explosions on Rockhopper penguins *Eudyptes chrysocome*. Cormorant.

[33] Cooper J (1982). Methods of reducing mortality of seabirds caused by underwater blasting. Cormorant.

[34] Wever EG, Herman PN, Simmons JA, Hertzler DR (1969). 1969. Hearing in the Black-footed penguin as represented by cochlear potentials. PNAS.

[35] Pichegru L (2012). Industrial fishing, no-take zones and endangered penguins. Biol. Conserv..

[36] Pichegru L, Grémillet D, Crawford RJM, Ryan PG (2010). Marine no-take zone rapidly benefit endangered penguin. Biol. Lett..

[37] BirdLife International. [*Spheniscus demersus*] *IUCN Red List of Threatened Species*. https://www.iucnredlist.org. Downloaded15 March 2015.

[38] African Penguin Biodiversity Management Plan, 2013. Department of Environmental Affairs, South Africa.

[39] Malme, C. I., Würsig, B., Bird, J. E. & Tyack, P. L. [Observations of feeding gray whale responses to controlled industrial noise exposure] *Port and ocean engineering under Arctic conditions*, *Volume* II [Sackinger, W. M., Jeffries, M. O., Imm, J. L. & Treacy, S. D. (eds.)] [55–73] (Fairbanks: University of Alaska, Geophysical Institute, 1998).

[40] Gailey G, Würsig B, McDonald TL (2007). Abundance, behavior, and movement patterns of western gray whales in relation to a 3-D seismic survey, Northeast Sakhalin Island, Russia. Environ. Monit. Assess..

[41] Fewtrell JL, McCauley RD (2012). Impact of air gun noise on the behaviour of marine fish and squid. Mar. Poll. Bull..

[42] McInnes AM (2015). Recreational fish-finders - an inexpensive alternative to scientific echo-sounders for unravelling the links between marine top predators and their prey. PLoS ONE.

[43] McInnes AM (2017). Small pelagic fish responses to fine scale oceanographic conditions – implication for the endangered African Penguin. Mar. Ecol. Progr. Ser..

[44] Popper AN, Hastings MC (2009). The effects of human-generated sound on fish. Integr. Zool..

[45] Dooling, R. J. [Auditory Perception in Birds] *Acoustic communications in birds* [Kroodsma, D., Miller, E. H. & Ouellet, H. (eds)] [95–130] (Academic Press, New York, 1982).

[46] Johansen, S. *et al*. [In-air and underwater hearing in the Great Cormorant (*Phalacrocorax carbo sinensis*)] *The Effects of Noise on Aquatic Life* II [Popper, A. N. & Hawkins, A. (eds.)] [505-512] (Springer Science + Business Media, New York, 2016). 10.1007/978-1-4939-2981-8_61 26610998

[47] Anisimov VD (1976). The morphology of the middle ear in penguins in Russian Vestn. Mosk. Univ. Ser. VI. Biol. Pochvoved.

[48] Bayne EH (2008). Impacts of chronic anthropogenic noise from energy-sector activity on abundance of songbirds in the boreal forest. Conserv. Biol..

[49] Melvin EF, Parrish J, Conquest LD (1999). Novel tools to reduce seabird bycatch in coastal gillnet fisheries. Conserv. Biol..

[50] Favaro, L., Gamba, M., Alfieri, C., Pessani, D. & McElligott, A. G. Vocal individuality cues in the African penguin (*Spheniscus demersus*): a source-filter theory approach. *Sci*. *Rep*. 1–12 (2015). 10.1038/srep17255 PMC4658557 26602001

[51] Jouventin, P. *Visual and vocal signals in penguins*, *their evolution and adaptive characters* [Paul Parey, Berlin, 1982].

[52] Favaro L, Ozella L, Pessani D (2014). The vocal repertoire of the African Penguin (*Spheniscus demersus*): structure and function of calls. PLoS ONE.

[53] Broni SC (1985). Social and spatial foraging patterns of the jackass penguin, *Spheniscus demersus*. South Afr. J. Zool..

[54] Ryan PG, Edwards L, Pichegru L (2012). African penguins *Spheniscus demersus*, bait balls and the Allee effect. Ardea.

[55] McInnes AM, McGeorge C, Ginsberg S, Pichegru L, Pistorius PA (2017). Group foraging increases foraging efficiency in a piscivorous diver, the African Penguin. Proc. R. Soc. B.

[56] Frost PGH, Shaughnessy PD, Semmelink A, Sketch M, Siegfried WR (1975). The response of Jackass Penguins to Killer Whale vocalisations. S. Afr. J. Sci..

[57] Smith, M. E. [Relationship between hair cell loss and hearing loss in fishes] *The Effects of Noise on Aquatic Life* II [Popper, A. N. & Hawkins, A. (eds)] [1067–1074] (Springer Science + Business Media, New York, 2016). 10.1007/978-1-4939-2981-8_132 26611069

[58] de Soto NA (2013). Anthropogenic noise causes body malformations and delays development in marine larvae. Sci. Rep..

[59] Otten W (2004). Acute and long term effects of chronic intermittent noise stress on hypothalamic–pituitary–adrenocortical and sympathoadrenomedullary axis in pigs. Anim. Sci..

[60] Anderson PA, Berzins IK, Fogarty F, Hamlin HJ, Guillette LJ (2011). Sound, stress, and seahorses: the consequences of a noisy environment to animal health. Aquaculture.

[61] Crawford RJM (2011). Collapse of South Africa’s penguins in the early 21st century: a consideration of food availability. Afr. J. Mar. Sci..

[62] Costa, D. P. *et al*. [A bioenergetics approach to understanding the population consequences of disturbance: elephant seals as a model system] *The Effects of Noise on Aquatic Life II* [A.N. Popper, A. N. & Hawkins, A. (eds)] [161–169] (Springer Science + Business Media, New York, 2016). 10.1007/978-1-4939-2981-8_19 26610956

[63] Boersma PD, Rebstock GA (2009). Foraging distance affects reproductive success in Magellanic penguins. Mar. Ecol. Prog. Ser..

[64] Pichegru L (2013). Increasing breeding success of an Endangered penguin: artificial nests or culling predatory gulls?. Bird Conserv. Inter..

[65] Sherley RB (2015). Bottom-up effects of a no-take zone on endangered penguin demographics. Biol. Lett..

[66] Montgomery, J. C., Jeffs, A., Simpson, S. D., Meekan, M. & Tindle, C. [Sound as an orientation cue for the pelagic larvae of reef fishes and decapod crustaceans] *Advances in Marine Biology* [Sims, D. W. & Southward, A. (eds)] [143–196] (Academic Press, 2006). 10.1016/S0065-2881(06)51003-X 16905427

[67] Slabbekoorn H (2010). A noisy spring: the impact of globally rising underwater sound levels on fish. Trends Ecol. Evol..

[68] Vermeij MJA, Marhaver KL, Huijbers CM, Nagelkerken I, Simpson SD (2010). Coral larvae move toward reef sounds. PLoS One.

[69] Nelms SE, Piniak WED, Weir CR, Godley BJ (2016). Seismic surveys and marine turtles: an underestimated global threat?. Biol. Conserv..

[70] Andriguetto-Filho JM, Ostrensky A, Pie MR, Silva UA, Boeger WA (2005). Evaluating the impact of seismic prospecting on artisanal shrimp fisheries. Cont. Shelf Res..

[71] Parry GD, Gason A (2006). The effect of seismic surveys on catch rates of rock lobsters in western, Victoria, Australia. Fish. Res..

[72] Jepson PD (2003). Was sonar responsible for a spate of whale deaths after an Atlantic military exercise?. Nature.

[73] Wilson RP (1985). The Jackass Penguin (*Spheniscus demersus*) as a pelagic predator. Mar. Ecol. Progr. Ser..

[74] van Eeden, R. B. The foraging ecology of African penguins in relation with ocean physical processes and prey availability. MSc thesis, University of Cape Town (2012).

[75] R Core Team. *R: A Language and Environment for Statistical Computing* (Vienna, Austria) Retrieved from https://www.r-project.org/ (2015).

[76] Hijmans, R. J., Williams, E. & Vennes, C. Spherical trigonometry. R package ‘Geosphere’. https://cran.r-project.org/web/packages/geosphere/index.html (2015).

[77] Bates D, Maechler M, Bolker BM, Walker S (2015). Fitting Linear Mixed-Effects Models using {lme4}. J. Stat. Soft..

[78] Lund, U. & Agostinelli, C. Circular statistics. R package ‘circular’. https://cran.r-project.org/web/packages/circular/index.html (2013).

